# Diagnostic, Therapeutic and Prognostic Potential of Pigment Epithelium-Derived Factor in Cancer

**DOI:** 10.3390/ijms26136004

**Published:** 2025-06-23

**Authors:** Crispin R. Dass, Joshua Dass

**Affiliations:** 1 Curtin Medical School, Curtin University, Bentley 6102, Australia; joshua.dass@curtin.edu.au; 2 Curtin Medical Research Institute, Curtin University, Bentley 6102, Australia; 3 Faculty of Pharmacy, Silpakorn University, Nakhon Pathom 73000, Thailand; 4 Radiation Oncology, Sir Charles Gairdner Hospital, Nedlands 6009, Australia

**Keywords:** PEDF, biomarker, cancer, metastasis, metabolism, oxidative stress

## Abstract

This review highlights recent findings on the versatile serpin protein, pigment epithelium-derived factor (PEDF), in relation to cancer diagnosis, treatment and prognosis. PEDF was initially discovered in the eye but has since been reported to be relevant to various biological roles in the body, and when awry, to clinically lead to various disease states such as neoplasia. At the preclinical stage, potent effects have been reported in studies focussing on apoptosis, metastasis, oxidative stress, immune stimulation and metabolism. Apart from full-length proteins, short peptides based on PEDF have shown promise against cancer. For diagnosis and prognosis, PEDF levels in tumour specimens or in circulation have the potential to serve as biomarkers, most probably in combination with other biomarkers of cancer initiation and progression. Lastly, this review discusses the growing list of studies that point out the perceived pro-cancerous effects of PEDF, though this is clearly outweighed by the anticancer publications. Thus, this review provides a comprehensive and balanced listing of the oncological studies associated with this protein to date, drawing conclusions on whether this potent antiangiogenic protein and its peptides can be used in the future for better cancer treatment, especially against metastasis.

## 1. Introduction

Pigment epithelium-derived factor (PEDF) is a 418 amino acid long protein with various biological effects in the body, including anticancer activity, which is linked to its pro-differentiation, pro-apoptotic, anti-angiogenic, and anti-metastatic activities, amongst other biological and physiological properties. One of the largest body of works on this protein deals with its anticancer properties, the focus of this paper.

The concept of associating PEDF to tumorigenesis or tumour progression was proffered in 1999 [1]. In that study, treatment with PEDF (known to be referred to as early population doubling level cDNA-1, EPC-1, protein back then) had no effect on endometrial stromal fibroblast (ESF) proliferation but did inhibit proliferation of endometrial carcinoma cells in a time- and dose-dependent manner in culture. As ESFs age in vitro, it was noted that the level of PEDF mRNA declines, commensurate with the level of protein secreted.

For a better understanding of the background to PEDF structure and biology, readers are referred to other reviews [2,3]. In our lab and those of select others, while effects of the serpin on cellular proliferation have been minimal, it has been found that PEDF regulates several metastatic processes angiogenesis inhibition, apoptosis, extracellular matrix (ECM) degradation, and epithelial-to-mesenchymal transition (EMT) dampening [2].

Thus, this review looks at the status quo of this serpin in cancer treatment, drawing upon extensive work carried out at the preclinical stage, and the increasing body of work linking this protein with diagnostic and prognostic potential clinically.

## 2. Preclinical Evidence

### 2.1. Metastasis

*Prostate:* PEDF inhibits the migration of castration-refractory prostate cancer (CRPC) cells [4]. In vivo, PEDF decreases PC3 and CL1 tumours growth in vivo. In the CL1 orthotopic model, tumour take with metastases was found in all animals, though PEDF prolonged the median survival of tumour-bearing mice. Accordingly, PEDF delayed the emergence of skeletal-related events in orthotopic intratibial xenografts. Metastases were reduced in combination PEDF + docetaxel (low dose) treatment compared to solo treatments, suggesting that the combination therapy delayed metastases formation.

PEDF induces the motility of macrophages towards prostate cancer spheroids [5]. In co-culture, PEDF increased phagocytosis of PCa cells through apoptosis, possibly via superoxide production in macrophages. Conditioned media (CM) from macrophages exposed to PEDF was pro-apoptotic in tumour cells suggesting that ROS may be involved in this cell death.

The vacuolar H+-ATPases (V-ATPases) have been linked with cancer metastasis [6]. When reintroduced, PEDF (which is commonly downregulated in prostate cancer cells) decreased the rate of proton (H+) fluxes (function of V-ATPases) in metastatic prostate cancer cells without affecting it in non-metastatic counterparts [7]. Caveolin-1 (Cav) which may be overexpressed in prostate cancer, and promotes its growth and metastasis, Cav increased DNA synthesis in prostate cancer cells, which was suppressible by PEDF [8].

The diabetic drug metformin inhibits the proliferation and colony formation of prostate cancer cells in a dose- and time-dependent manner [9]. It also suppresses migration and invasion, induces apoptosis of prostate cancer cells, and reduces human PC3 tumour growth in nude mice. Relevant to this review, metformin efficacy is associated with higher PEDF expression in both prostate tumour cells and tissue, and this could be a mechanism for its anti-metastatic activity.

PEDF protects against the doxorubicin toxicity in tissues of the heart, small intestine and testes [10]. In the same study, PEDF inhibited proliferation and promoted apoptosis in human prostate and breast cancer cells, which metastasise to the bone. Caspase-2 was activated in both tumour cell types by PEDF. In murine models of prostate and breast cancer propagation in bone, PEDF significantly reduced tumour volumes at the primary site. When combined with the frontline anti-bone-resorption drug zoledronic acid, PEDF reduced breast tumour metastasis to the bone, and was intriguingly able to preserve the quality of bone better than the combination therapy in a breast tumour model.

*Breast:* PEDF suppresses migration and invasion in SKBR3 (luminal) breast cancer cells and leads to morphologic and molecular changes towards EMT [11]. A reduction in PEDF promotes the mesenchymal phenotype, whereas PEDF was shown to channel cells to an epithelial phenotype. However, in a different study [12], PEDF inhibited breast cancer cell migration and invasion by downregulating MMP2 and MMP9 via p-ERK and p-AKT signalling pathways, and fibronectin, but in contrast, failed to impose itself on EMT. This study demonstrated that PEDF dampened breast cancer metastasis by downregulating fibronectin via the laminin receptor/AKT/ERK pathway. In invasive ductal breast cancer (IDC), the five-year survival rate was higher for patients with PEDF-positive tumours [13]. This team also found that PEDF associated with EMT-related genes, suggesting that it may dampen EMT.

The ability of PEDF to attenuate metastatic markers such as matrix 1 metalloproteinase (MT1-MMP) and focal adhesion kinase (FAK) has been shown in our lab in various types of cancers, including chondrosarcoma [14], breast and prostate cancer [15]. In breast cancer, Dox + PEDF was found to reduce glucose uptake in MDA-MB-231 cells but increased it in MCF-7 cells [16]. While PEDF hindered tumour cell migration from tumour spheroids, the combination was most inhibitory. Intriguingly, PEDF elevated phosphorylated IRS-1 (p-IRS1) levels in both cell lines, and in both, PEDF and combination treatment downregulated levels of p-Akt. This highlights the shift towards metabolic studies to better understand and manage cancers.

PEDF can decrease phosphorylated-nuclear factor-kB p65 subunit (p-NFkB-p65), tumour necrosis factor- (TNF), C-X-C chemokine receptor type-4 (CXCR4), and urokinase plasminogen activator receptor (uPAR) in ER+/HER2- breast cancer cells under post-menopausal oestrogen concentrations [17]. In triple negative breast cancer (TNBC) cells, it reduced pNFkB-p65 and uPAR expression under pre-menopausal oestrogen levels [18]. We identified a plausible regulatory relationship between p-NFkB-65 and PEDF, which was breast cancer subtype-specific and differentially regulated by menopausal oestrogen conditions. PEDF reduced cellular viability, while combined PEDF and NFkB-p65 inhibition proved superior in reducing breast cancer cell colony formation.

*Osteosarcoma:* One of the largest body of work in the area of metastasis and PEDF came through the University of Melbourne/St Vincent’s Health in the mid-2000s. It was demonstrated that PEDF upregulates collagen I, heat shock protein 47 (HSP47) and membrane type 1 matrix metalloproteinase (MT1-MMP), while downregulating MMP-2 in osteosarcoma cells in vitro [19]. This has significant implications on the ability of osteosarcoma cells to migrate and metastasise, with reduction in VEGF levels, which has been noted previously in numerous in vivo studies of spontaneously metastasising disease [20,21,22,23,24,25].

*Colorectal:* A PEDF-plasmid DNA-loaded liposome for gene therapy for metastatic colorectal cancer (CRC) was formulated via an iRGD peptide, and demonstrated to inhibit invasion and migration of, and promote apoptosis in, cultured CRC cells [26]. In a murine model of CRC, the PEDF-DNA-liposome reduced metastatic burden in the lung and prolonged survival time. In another study using a CRC model, PEDF decreased tumour MVD, increased macrophage infiltration, and improved response to metronomic cyclophosphamide (CPA) dosing against pulmonary metastasis [27].

*Cervical:* liposomes targeted to folate receptor α (FRα) (FLPs) were used to encapsulate PEDF plasmid and were found to inhibit growth of cultured HeLa cells and human umbilical vein ECs (HUVEC cells) [28]. It also suppressed adhesion, invasion and migration of cultured HeLa cells. In a metastatic model of cervical cancer, FLP/PEDF administered intraperitoneally had a profound anti-tumour effect probably due to the upregulated PEDF. FLP/PEDF reduced MVD, inhibited proliferation, and promoted apoptosis of tumour cells in vivo.

*Bladder:* Levels of miR-93 in the urine are elevated in patients with bladder cancer than in their healthy counterparts [29]. This miRNA has a binding site on PEDF and its inhibition suppressed the proliferation and invasion of bladder cancer cells, by increasing PEDF levels [30].

*Kidney:* A direct interaction between PEDF and miR-93-3p has been confirmed [31]. Akin to miR-93-3p inhibition, PEDF overexpression induced tumour cell apoptosis and inhibited their migration and invasion. siRNA to PEDF reversed the effects of this miRNA’s inhibition in clear cell renal cell carcinoma cultured cells.

*Lung:* The above findings reverberated those of another study [32] showing that overexpression of PEDF reduced non-small cell lung cancer (NSCLC) invasion and migration, while promoting cell adhesion. PEDF knockdown opposed these effects. Furthermore, exosomes from NSCLC cells treated with recombinant PEDF had reduced capacity for promoting cancer cell motility and invasion.

*Neurofibromatosis:* Neurofibromatosis type 1 (NF1)-derived tumours are enriched in blood vessels, with the growth of NF1 tumours being angiogenesis-dependent [33]. PEDF inhibits proliferation and promotes apoptosis in cultured malignant peripheral nerve sheath tumour (MPNST; a type of NF1) cells [34]. In xenografts of MPNST cells in immunocompromised mice, PEDF suppressed MPNST tumour burden, largely as a result of antiangiogenesis.

*Melanoma:* PEDF inhibited metastasis of uveal melanoma to the liver [35]. Mice expressing PEDF exhibited significantly lower MVD. Normal dermal fibroblasts with high PEDF expression attenuated melanoma growth and angiogenesis in mice, whereas fibroblasts with reduced PEDF promoted tumour formation [36]. Accordingly, PEDF KO mice were more susceptible to melanoma metastasis. The authors contended that their results demonstrate that PEDF maintains tumour-suppressive functions in CAFs to prevent pro-tumour conversion and shed some light on how melanoma cells hijack stromal PEDF to promote cancer progression.

The liver is the premier site for metastasis in over 75% of cases of uveal melanoma via the haematogenous route [37]. PEDF controls angiogenesis in a liver metastasis model of melanoma [38]. There were higher MVD values in PEDF KO animals when compared to control (wildtype) animals.

*Myeloma:* PEDF decreases vascular endothelial growth factor (VEGF), monocyte chemoattractant protein-1 (MCP-1), intercellular cell adhesion molecule-1 (ICAM-1) and plasminogen activator inhibitor-1 (PAI-1) mRNA levels in myeloma cells, through its signalling via the laminin receptor (LR) [39].

### 2.2. Apoptosis

In lung cancer cells, PEDF heightened the sensitivity to apoptosis by causing the translocation of Fas protein to the cell membrane [40]. Upregulation of FasL by PEDF was mediated by p53, which was regulated in turn by peroxisome proliferator-activated receptor gamma (PPAR-γ). The combined treatment of low dose cisplatin plus adeno-associated virus (AAV)-delivered PEDF prolonged the survival of mice and effected suppression of de novo tumour vascularisation and induced apoptosis in tumour tissue in vivo [41]. PEDF also protected the mice from cisplatin-related toxicity. AAV-PEDF + hyperthermia suppresses tumour growth in a murine model of subcutaneous fibrosarcoma [42]. The combination treatment inhibits angiogenesis and induces apoptosis in tumour tissues. Hyperthermia also increases PEDF expression in vivo.

Systemic administration of an adenoviral vector expressing PEDF (Ad-PEDF) in a liposomal formulation causes marked suppression of B16-F10 tumour growth, and provoked apoptosis in B16-F10 melanoma cells and inhibited pulmonary metastases [43]. PEDF drives apoptosis of ovarian cancer cells [44]. PEDF is repressed in ovarian cancer cells compared to their normal counterparts. PEDF overexpression in tumour cells inhibited tumour growth in a chorioallantoic membrane model.

Infantile haemangioma, a common vascular tumour, occurs in approximately 5–10% of infants [45]. In haemangioma-derived endothelial cells, increasing the PEDF/VEGF ratio inhibits proliferation, migration, and tube formation in these cells, and promotes apoptotic cell death [46]. In mice, PEDF has a growth-suppressive and proapoptotic effect on lung tumours. Accordingly, in vitro, PEDF apparently induced apoptosis in lung cancer cells, mostly through the Fas-L/Fas death signalling pathway [40]. PEDF engages both FAP-1 and p53 to promote translocation of Fas to the plasma membrane. PEDF also upregulates Fas-L via p53, which in turn is regulated by PPAR-γ.

PEDF mRNA level is significantly decreased in ovarian cancer (OvCa) and correlates with OvCa progression and tumour-associated macrophage markers [47]. OvCa tumours which overexpress PEDF show suppressed growth and increased apoptotic rate. PEDF promotes macrophage polarisation in OvCa tumours towards an M1 subtype, via activation of adipose triglyceride lipase (ATGL) and extracellular-regulated kinase 1/2 (ERK1/2) signalling.

PLGA nanoparticles loading both PEDF gene and paclitaxel [48] disrupted tube formation in primary HUVECs. Nanoparticles enhanced antiangiogenic activity in the transgenic zebrafish and alginate-encapsulated tumour cell models. NPs achieved a markedly higher antitumour efficacy in the C26 tumour-bearing mice model, as displayed by inhibition of tumour cell proliferation and angiogenesis and induction of apoptosis. In another study, PEDF plasmids were incorporated into nanoparticles (D-NPs) and demonstrated to have an excellent anticancer effect in both CT26 and A549 cells [49]. D-NPs also inhibited proliferation of HUVECs in vitro and inhibited tumour-induced angiogenesis in vivo. In a CT26 subcutaneous tumour model, D-NPs could achieve a significant antitumour activity with less MVD and heightened tumour cell apoptosis.

### 2.3. Oxidative Stress

PEDF blocked Wnt3a-directed induction of autophagy proteins in pancreatic intraepithelial neoplasia (PanIN) cells [50]. Autophagy inhibition was complemented by regulation of the oxidative stress enzymes, superoxide dismutase 2 (SOD2) and catalase. SOD2 expression was mediated by NFκB nuclear translocation induced by PEDF.

PEDF inhibits VEGF-mediated reactive oxygen species (ROS) generation, decreases anti-apoptotic and growth-promoting factor, increases myeloid cell leukaemia 1 (Mcl-1) expression, and reduces proliferation of multiple myeloma (MM) cells [51]. In addition, PEDF inhibits VEGF-mediated blockade of apoptosis in patient MM cells. Molecularly, it was found that PEDF blocks proliferation and survival of MM cells induced by VEGF, specifically suppression of p22phox, a plasma membrane-located component of nicotinamide adenine dinucleotide phosphate (NADPH) oxidase.

Clinically used anticancer drug doxorubicin increases levels of PEDF in an MDA-MB-231 human breast cancer cell line [52]. This occurrence was also observed in murine cardiac muscle tissue where PEDF levels increased as the dose of doxorubicin increased in vivo. PEDF boosted levels of reactive oxygen species (ROS) and glutathione (GSH) in MDA-MB-231.

In a breast cancer cell line, PEDF dose-dependently inhibited the ability of advanced glycation end-products (AGEs) to promote NADPH oxidase-driven superoxide generation, cytochrome b-245 β chain (gp91phox) and receptor for AGE (RAGE) mRNA, VEGF, and MMP-9 mRNA expression in MCF-7 cells, all dose-dependently inhibited by PEDF [53].

### 2.4. Immune Stimulation

A combined treatment of PEDF and cabazitaxel in a murine prostate cancer model led to disease stabilisation [54]. Furthermore, the combination therapy inhibited tumour cell migration and heightened tumoricidal activity of macrophages against prostate tumour cells.

PEDF and paclitaxel were co-encapsulated in a nanoparticle formulation [55]. Microtubules were stabilised and G2/M arrest occurred along with a high subG1 population. The CT26 model demonstrated a significant antitumour activity with the combination, including reduced MVD and increased tumour cell apoptosis. PEDF controlled anti-angiogenic responses, with high MVD in PEDF KO compared to NK-depleted and wildtype animals [38]. The myeloid lineage, comprising monocytes, macrophages, and myeloid-derived suppressor cells, was lesser in the absence of NK cells or PEDF.

### 2.5. Miscellaneous Anticancer Effects of PEDF

When adipose-derived mesenchymal stromal cells (ASCs) were transduced with PEDF-lentiviruses, and the conditioned medium exposed on PC3 cells, tumour inhibitory genes were expressed, suggesting that PEDF reduces the potential of tumour-promoting activity of unmodified ASCs [56].

PEDF inhibits non-small cell lung cancer (NSCLC) cell proliferation and viability and increases lactate dehydrogenase release and intercellular adhesion [57]. It also suppresses the expression and activation of microtubule-associated protein 1 light chain 3 and reduced development of autophagy, which complemented the reduction in NSCLC proliferation and cellular viability.

In the ER+ breast cancer cell line MCF-7, PEDF was noted to influence metabolism linked to amino acids, TCA cycle mediators, nucleotides, and lipids [58]. Results differed when cells were challenged under hyperglycaemic conditions. In a similar study, this time looking at the TNBC MDA-MB-231, potent effects on the Warburg effect were induced by PEDF [59]. The major markers that were attenuated (summarised in Table 1) provide hope that by targeting the Warburg pathway, effective cancer therapy may be possible.

Uterine fibroids (leiomyomas) are the most common benign lesions in women and can disrupt uterine function via several ways [60]. PEDF induced downregulation of VEGF, plus oestrogen receptors, whilst inhibiting cell proliferation. PEDF treatment of mice reduced fibroid growth.

When Lewis lung cancer cells were inoculated in immunocompromised mice were treated with radiation alone, PEDF alone, or PEDF combined with radiation, there was dramatic inhibition of tumour growth when tumours were irradiated between days 3 and 7 (microvessel normalisation window) after the PEDF administration commenced [61]. During this window, tumour blood vessels in the PEDF cohort were less tortuous and more uniform.

Phosphaplatins are platinum complexes ligated by diaminocyclohexane and pyrophosphate ligands (example, phosphaplatin platinum (IV) (RRD4) complex, and which have shown promising antitumour efficacy preclinically [62]. In breast cancer cells, RRD4 was able to upregulate PEDF, which the author believed contributed to the anticancer effect [63]. Again in breast cancer, PEDF inhibits tumour cell-derived and endothelial cell-derived angiogenesis by downregulating the hypoxia-inducible factor alpha (HIF-1α) protein [64], separating it from failed clinical antiangiogenic drug entities such as sorafenib, endostatin and bevacizumab.

### 2.6. Preclinical Studies Correlating PEDF Positively with Cancer Progression

There are a handful of papers portraying PEDF as a pro-cancerous factor. For instance, PEDF channels glioma stem cells towards cancer [65]. EGFRvIII promotes the PEDF expression and secretion via activation of signal transducer and activator of transcription 3, promoting self-renewal of glioma stem cells [66]. PEDF sustains glioma stem cell auto-renewal, and a subpopulation of such cells increased PEDF migrated into the corpus callosum, consistent with a tumour-like biology.

Blockade of PEDF restored tube formation and EC viability to levels observed in the extracellular fluid of non-cancerous tissue [67]. Moreover, in xenografted mice, the inhibition of angiogenesis, promotion of lymphangiogenesis, and the entrapment of intrahepatic cholangiocarcinoma cells in lymph nodes were shown to be PEDF-dependent. In gastric cancer, serum PEDF levels are significantly higher than that in both precancerous lesion and control groups [68]. Thus, PEDF does its traditional job, but another option opens up for cancer cell spread, perhaps a less malevolent one.

PEDF is overexpressed in oesophageal cancer cells and patient biopsies compared to normal tissue [69]. While PEDF enhanced mitosis and inhibited apoptosis, its knockdown inhibited proliferation and migration of cultured cancer cells. Inhibition of PEDF significantly reduces tumour growth and tumour size in vivo. The level of PEDF was found to be significantly higher in patients with prostate cancer than in those without [70]. PEDF was positively correlated with pathological grading (Gleason score), though its expression was only detected in few prostate cancer cells.

In a novel model of kidney-metastasising osteosarcoma, 143B cells were injected intracardially until the kidney-metastasising sub-cell line Bkid was established [71]. Here PEDF seems to be pro-cancerous as it mediates cancer cell extravasation by increasing the permeability of kidney and lung microvasculature acting via laminin receptor signalling. PEDF was the prime candidate gene identified for kidney metastasis. When inoculated intracardially, Bkid cells with PEDF knockdown failed to metastasise to the kidneys, and conversely, 143B with overexpressed PEDF injected into femur metastasised to the lungs and kidneys.

Thus, whilst a clear majority of cancer studies point out the direct and indirect anticancer effects of the serpin, especially in cultured cells where cancer cells are looked at in isolation, caution must be given to all studies looking at using this protein as a treatment agent against cancer, particularly in preclinical animal models of cancer.

### 2.7. PEDF Peptide-Specific Studies in Cancer

**Figure 1:**
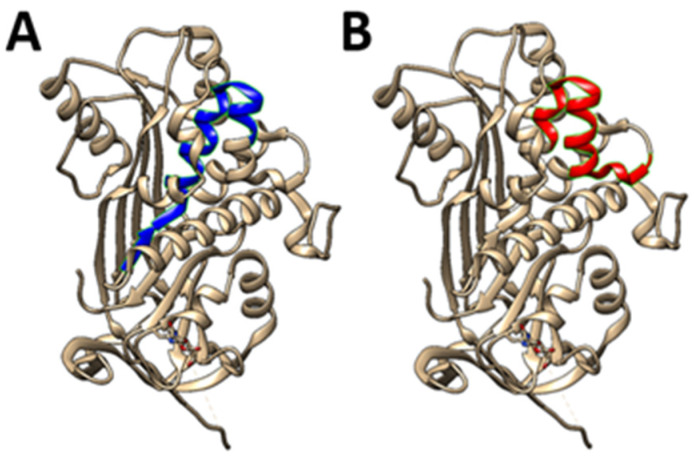
**PEDF-based 25-mer peptides.** Two unique short 25-mer peptides (**A**) **residues 78-102** (78 VLLSPLSVATALSALSLGAEQRTES102), and (**B**) **residues 90-114** (90 SALSLGAEQRTESIIHRALYYDLIS 114), inhibit osteosarcoma growth and metastasis and like PEDF, support bone formation. In comparison, two other major PEDF peptides are the 34mer sequence (Asp44-Asn77), and the 44mer sequence (Val78–Thr121). *Advantages of using such short peptides rather than parent protein*: versatility may be problematic as parent protein has diverse (and perhaps conflicting) activities, the full-length protein is expensive, peptides should be more stable, several labs have designed short length peptides which show similar activity to full length protein, and peptides allow a degree of targeted activity.

One of the earliest application of PEDF peptides looked at two peptides (depicted in Figure 1) in an animal model of spontaneously metastasising osteosarcoma [22]. When OS cells were pre-mixed with peptides, then injected into the tibia, both primary growth and metastasis were affected. In a subsequent study, when one peptide was administered continuously via osmotic pumps, metastases were significantly reduced, suggesting a potential clinical utility of this peptide [24].

The death receptor pathway can be activated by the 34-mer peptide through FasL and caspase-8 in both tumour tissues and in vitro [72]. As NF-κB and PPARγ are crucial transcription factors for FasL expression, when tested, the peptide upregulated PPARγ but not NF-κB. 34-mer has stronger hydropathicity and more interactions with laminin receptor (LR) than the 44-mer variant. LR block abolished PPARγ and FasL upregulation by the 34-mer peptide. Interestingly, it was found that PEDF34 pro-apoptosis induction was similar in both ECs and tumour cells.

PEDF delivery in mice reduced LRP6 activation [73]. In human hepatocellular carcinoma (HCC) cells, PEDF silencing increased activated LRP6 and β-catenin, while the 34-mer PEDF peptide decreased LRP6 activation and β-catenin signalling, reducing Wnt target genes. Finally, PEDF KO mice maintained on a Western diet progressed to sporadic and well-differentiated HCC. In corroboration, human HCC specimens had reduced PEDF staining compared to hepatocytes.

A triple phosphomimetic version of PEDF, called EEE-PEDF, induces endothelial cell apoptosis through caspase-3, and inhibits migration of the EC much better than the wildtype PEDF sequence [74]. Acute and chronic exposure of CRC cell lines to c-terminus-based PEDF peptides lowered drug-resistance to conventional drugs such as oxaliplatin [75]. After xenograft transplantation, the peptides reduced resistance to cytotoxics, plus metastasis was reduced.

### 2.8. Translational Evidence

*Pro-cancer:* high expression of PEDF is associated with shorter overall survival in HCC patients [76]. Forced PEDF expression enhanced HCC cell aggressive behaviour in vitro and in vivo, whereas silencing it did the opposite. PEDF expression led to changes in cell morphology, propelling cells down the EMT pathway, and promoting EMT-related markers via ERK1/2 signalling pathway. Clinically, in HCC specimens, PEDF correlates with subcellular localisation of laminin receptor (LR), and high expression of PEDF and LR aligned with a weaker prognosis. ATGL was not implicated in this study to drive tumorigenesis and progression. The study did not differentiate as to the stage of site of collection of the specimens in the cohort of patients disabling attempts to elucidate if any tissue-specific effects were at play in the pro-cancerous effects of PEDF.

PEDF negatively correlates with tumour cell invasion and metastasis in oesophageal squamous cell carcinoma [77]. A positive correlation between PEDF expression and nodal stage and TNM (tumour/nodal/metastasis) stage were noted. The high expression of PEDF seems to be an unfavourable association with OSCC patient overall survival. Activation of the MAPK/ERK signalling pathway was promoted by PEDF in its role in inducing tumour cell motility and EMT. It has been suggested that secretion of PEDF by pancreatic cancer cells may lead to sprouting of the nerves toward cancer structures and neural invasion [78].

It remains to be fully proven that PEDF is a precancerous protein, as a majority of clinical findings report an association, sometimes with some preclinical data. However, moving forward, it pays for PEDF researchers to be aware of this dubiety, and every attempt should be made to monitor this and either rule out or confirm its presence. PEDF is a known cytoprotective agent, and we believe that the high levels seen in certain cancer patients may well be a part of this defensive role it plays in the body.

*Anticancer:* a comprehensive search for the studies on PEDF expression in 14 top-ranked cancers with the highest incidence, and meta-analysis was performed to investigate whether PEDF associates with staging, grade, size, lymph node and distal spread [79]. A Kaplan–Meier curve was prepared to gauge the effect of PEDF expression on patient prognosis. Decreased PEDF associates with higher TNM staging, larger tumour size, increased propensity for lymph node invasion and advanced pathological grade. Low PEDF protein levels in tumour tissue correlates to shorter overall survival.

Expression of PEDF was diminished in human nasopharyngeal carcinoma (NPC) tissues [80]. PEDF knockdown induced EMT in low metastatic NPC cell lines, while PEDF overexpression restored epithelial phenotype in aggressive cell lines. PEDF inhibition drove NPC cells towards metastasis in vivo. This study demonstrated that LRP6, GSK3β, β-catenin signal pathway rather than the AKT, GSK3β pathway was involved in the effects of PEDF on EMT. In another NPC study, PEDF was diminished and associated with clinicopathological and EMT features [80].

PEDF expression was downregulated in a study looking at colorectal cancer (CRC) [81]. Treatment with PEDF decreased the rate of CRC cell migration and invasion, and increased cellular adhesion in various CRC cell lines examined. Additionally, PEDF may be an osteogenic factor in CRC with osseous metaplasia [82]. There is low PEDF expression in human NPC which positively correlates with poor prognosis and negatively with lymph MVD [83]. PEDF inhibits lymph-associated spread of NPC in animal models of disease. It does this by inhibiting proliferation, migration, and tube formation of lymphatic endothelial cells, and promotion of cell death via apoptosis. On a molecular level, PEDF reduces expression and secretion of vascular endothelial growth factor C (VEGF-C) from NPC cells through nuclear factor-κB (NF-κB) signalling.

PEDF expression is lower in human pancreatic cancer compared to non-malignant tissue [84]. PEDF, together with vascular cell adhesion molecule 1 (VCAM1) and hepatocyte growth factor activator (HGFA), were identified as promising biomarkers for extramedullary MM (EMM), demonstrating reasonable accuracy in distinguishing EMM patients from MM patients [85]. In HCC, histological grade and portal vein invasion was corelated with LR, while high levels of PEDF in HCC associates with lack of portal vein invasion [86]. There was a lack of elevated PEDF expression in cases with more than 5% fatty degeneration in the background liver tissue.

Although osteogenesis imperfecta (OI) is characterised by bone fragility and deformities, and other connective tissue deficiencies, it is not linked to greater risk of skeletal tumours. A report on an adult with OI in which a deletion in exon 8 of PEDF noted that the patient presented popcorn calcification in both femoral epiphyses, with one of them being diagnosed as chondrosarcoma [87].

PEDF expression is consistently decreased in aggressive melanoma, in contrast to higher levels in nevi and melanoma in situ [88]. PEDF was lost in thicker melanomas, and correlated inversely with depth of invasion and distal metastasis. PEDF levels are higher in patients with lung adenocarcinoma compared to patients with the more aggressive variant, lung squamous cell carcinoma [26].

In metastatic breast patients diagnosed as being ER+, HER2-, or TNBC, cytoplasmic and membrane PEDF are lower in bony metastases compared to primary tissue [17,18]. Nuclear PEDF staining was higher in secondary compared to primary TNBC, and higher membrane PEDF in metastatic tissue had improved disease-free interval. In contrast, nuclear staining of PEDF was lower in bony metastases compared to primary ER+/HER2- breast cancer in post-menopausal patients.

Decreased PEDF expression was noticed in clinical tumour tissue compared with healthy urothelium [89]. Lower PEDF expression was related to higher tumour grade. Expression of PEDF was correlated with MVD negatively in cancerous tissue. Breast cancer lesions > 2 cm had lower peritumoral stromal expression of PEDF than smaller tumours [90]. In cases with lymph node involvement, PEDF levels were lower in peritumoral stroma compared with node-negative cases.

In locally advanced rectal carcinoma (LARC) patients, PEDF is negatively correlated with tumour differentiation, and increasing tumour stages [91]. Overexpression of PEDF in aggressively metastatic cells enhances radiosensitivity and dampens migration and invasion in vitro. PEDF suppresses tumour growth in an animal model of disease. PEDF performs these functions via activating P53. In cultured bladder cancer cells, PEDF expression negatively correlates with AR expression, and while androgen upregulates AR, it does the converse to PEDF. PEDF level was significantly negatively correlated with EMT [92].

PEDF expression levels are significantly correlated with lymph node metastasis, extrathyroid invasion, a high TNM stage, and tumour size in papillary thyroid carcinoma, PTC [93]. However, the studies closing statement sums it all up: PEDF plays a role in the progression of PTC but is anti-angiogenic by attenuating the HIF1α/VEGF pathway, thus essentially inhibiting the metastasis of PTC in the longer term.

PEDF levels in blood and association with clinical diagnosis and prognosis.

Serum levels of PEDF were evaluated in liver cirrhosis patients and it was noted that PEDF levels were higher in the cirrhotic cohort than in the control group [94]. In patients with alcoholic or mixed (alcoholic and viral hepatitis-related) cirrhosis, PEDF was higher in the serum than in other patients. Of note, in patients with viral hepatitis-related cirrhosis, elevated PEDF was noted in those with HCC than those without, moving the authors to speculate that it could be used as an auxiliary biomarker for disease especially in those with low α-foetoprotein.

In a study searching for reliable markers for CRC [95], decreased PEDF in serum correlated with liver metastasis and poor disease-free survival (DFS) and overall survival (OS). Thus, it was deemed to be a potential prognostic marker for CRC. PEDF is underexpressed in sera exosomes of osteosarcoma patients exhibiting poor chemotherapeutic response when compared with good chemotherapeutic response [96]. Thus, exosomal RNAs such as PEDF can discriminate good and poor chemotherapeutic response for osteosarcoma treatment.

A higher expression of PEDF was noted in the sera of oral squamous cell carcinoma patients who chewed tobacco compared to healthy volunteers [97]. In OvCa patients, PEDF levels in ascites and serum were found to be higher in advanced disease than benign tumours [98]. PEDF correlates with early recurrence of OC patients, which point to the serpin being a possible prognostic biomarker in OC.

## 3. Conclusions

**Figure 2:**
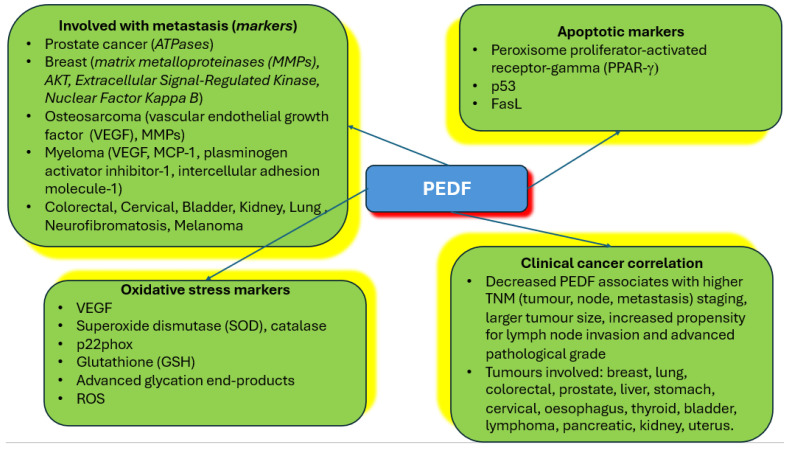
**Summary of PEDF relationships to cancer and major markers uncovered.** The figure summarises the types of tumours where PEDF is implicated in metastasis, markers of metastasis that are engaged by PEDF, apoptotic markers affected by PEDF in cancer cells, oxidative stress markers activated by PEDF in cancer cells, and correlation of PEDF at the protein level with various types of cancers found clinically.

The past decade of research into PEDF has uncovered the potential of this serpin to serve as a biomarker for certain types of cancers. This potential may be greater if such an association with more cancers is studied both at the diagnostic and prognostic levels. This correlation stems from the past two decades devoted to determining the anticancer effects of this protein (Figure 2) and its peptides in preclinical studies. Certainly, the significant effects PEDF has on cancer metastasis stand out, and is perhaps the aspect which can be developed further towards the clinical evaluation of the protein’s capacity in treatment of cancer patients. A developing area for PEDF in cancer is metabolomics analysis, as once we know which markers are upregulated or downregulated, researchers can attempt to focus and target specific metabolic pathways, even with changes in dietary habits, leading to tailor-made and personalised therapy. This is linked with the protein’s anti-oxidative properties, which may explain why it is overexpressed in later-stage cancers. Certainly, more studies are therefore warranted as the attempts to test this protein and its peptides clinically against cancer gathers momentum.

## Figures and Tables

**Table 1:** A summary of major metabolic biomarkers attenuated in breast cancer cells by PEDF.

Metabolite (Polar)	Up-/Down-Regulated	Pathway
Adenosine	Down	Nucleoside
Cysteine	Up	Amino acid
Glucose-6-phosphate	Down	Glycolysis
Glutamic acid	Up	Amino acid
Glycine	Down	Amino acid
Homocysteine	Down	Amino acid
Lactic acid	Down	Warburg
Methylmalonic acid	Down	Amino acid breakdown
Pyruvic acid	Down	Glycolysis and Krebs cycle
Ribulose-5-phosphate	Up	Pentose phosphate/Calvin cycle
Stearic acid	Down under hyperglycaemia	Lipid
Uracil	Down	Nucleoside
**Metabolite (Non-polar)**	**Up-/down-regulated**	**Pathway**
Cholesterol	Down under hyperglycaemia	Lipid
Palmitic acid	Down under hyperglycaemia	Lipid
Palmitoleic acid	Down	Lipid
Phosphoethanolamine	Down	Phospholipid precursor
Oleic acid	Down	Lipid
